# Asian “Guan” Parenting and Life Satisfaction Among Adolescents in Malaysia: The Mediating Role of Filial Piety

**DOI:** 10.3389/fpsyg.2021.746540

**Published:** 2021-11-26

**Authors:** Sarvarubini Nainee, Soon-Aun Tan, Chee-Seng Tan, Su-Wan Gan, Jo-Pei Tan

**Affiliations:** ^1^Department of Psychology and Counselling, Faculty of Arts and Social Science, Universiti Tunku Abdul Rahman, Perak, Malaysia; ^2^Faculty of Health, Psychology and Social Care, Manchester Metropolitan University, Manchester, United Kingdom

**Keywords:** adolescents, filial piety, guan parenting, life satisfaction, Malaysia, statistical mediation, well-being

## Abstract

Literature on adolescent development has shown that parenting practices have positive relationships with adolescents’ life satisfaction. Adolescents’ life satisfaction improves when they have parents low in psychological control who uphold reciprocal self-disclosure in their communication. Guan parenting was found to correlate positively with adolescents’ development. Therefore, it is methodologically important to replicate the investigation on the relationship between adolescents’ life satisfaction and Guan parenting. Literature suggests that filial piety is shaped by parenting practices and adolescents who perceived intense parental concern, care, and involvement tend to uphold filial piety and express gratitude toward parents which may promote the adolescents’ life satisfaction. In this study, mediation analysis was done to elucidate the relationship among parents’ guan parenting style, filial piety, and life satisfaction on 606 adolescents (M_age_=15.07; SD_age_=1.03; 52.1% females) in Malaysia. The adolescents were sampled through cluster sampling, and data were collected using self-administered questionnaires. The results showed positive relationship between paternal and maternal guan parenting with filial piety and adolescents’ life satisfaction. Greater parents’ filial piety was linked to higher life satisfaction among adolescents. Findings from the mediation models indicated the association among guan parenting with filial piety, gratitude toward parents, and higher life satisfaction. The findings also offered empirical evidence to the underlying mechanism of how guan parenting could affect adolescent life satisfaction *via* the mediating role of filial piety. The findings also supported the importance of culture-infused parenting in inculcating adolescents’ filial piety besides establishing its link to life satisfaction in Asian families.

## Introduction

### Life Satisfaction Among Adolescents

Life satisfaction plays a key role in adolescents’ achievement of developmental tasks and fulfilling social roles. In recent years, there has been increased attention and interest in the field of life satisfaction and quality of life especially among children and adolescents (Orben et al., 2019). A birth cohort study from Fergusson et al. (2015) revealed that life dissatisfaction was associated with mental health issues such as depression among youth.

Life satisfaction is an umbrella concept consisting of the contentment derived from the closest social circle which includes family (Huebner, 1991; Zhou et al., 2020). Life satisfaction also refers to an individual’s cognitive and subjective evaluation of well-being (Salimi, 2011; Tsurumi et al., 2021) that reflects the level of happiness or unhappiness. This may be related to the individuals’ perceptions toward their lives from satisfactory levels to life expectations, demands, and desires (Diener and Lucas, 1999). In short, high life satisfaction is linked to happiness and the achievement of a “good life,” whereas negative evaluations of one’s life mean the opposite (Proctor et al., 2008; Lado et al., 2021).

A recent study in Malaysia found that adolescents with a higher level of life satisfaction were reported to be less likely to engage in delinquent behavior (Mohamad et al., 2018). A past study also reported that adolescents’ satisfaction toward family and school life was a significant predictor of their internalizing and externalizing behavior (Haranin et al., 2007). For a nation with multi-ethnic people, cultural differences or lifestyles may impact one’s satisfaction toward life. Knowing that life satisfaction can be taken as a vital indicator of optimal outcomes for adolescents, it is therefore important to examine the impact of family factors like parenting styles and cultural factor like filial piety on adolescents’ life satisfaction.

### Guan Parenting

Chao (1994) proposed “guan” as an Asian parenting construct that entails structure and control for achieving the parental goal, investment, intense involvement, and physical closeness with children. Guan, also referred to as training, is defined as parental control in cultivating expected social values and guiding appropriate behavior of children (Chao, 1994; Luo et al., 2013). Choi et al. (2013) have described guan as the manifestation of affection and monitoring from parents. The concept of guan is also hypothesized to be distinct from dimensions of warmth and control (Chao, 1994) and may be particularly important for promoting positive outcomes in Asian children. A study by Stewart et al. (2002) that recruited samples from Hong Kong, Pakistan, and the United States reported that a greater level of guan parenting predicted a higher level of self-esteem and life satisfaction well-being of children. Theoretically, the transactional model of development discusses the importance of parenting practices on positive child development outcomes (Sanson et al., 2018). Empirical evidence also showed the effect of parenting practices on adolescents’ life satisfaction (Pérez-Fuentes et al., 2019; Kim and Choi, 2021). Over decades, previous studies have found a high level of guan parenting being associated with children’s life satisfaction (Stewart et al., 1999; Fok and Shek, 2011). However, little is known about the role of guan parenting in predicting the life satisfaction of Malaysian adolescents. Thus, it is crucial to examine the role of parenting in Malaysian families using a more culturally specific dimension like guan parenting.

Malaysia is a collectivist and multiracial society that consists of three major ethnic groups (i.e., Malays, Chinese, and Indians). Islamic, Chinese, and Indian cultures may exhibit universal parenting traits that share some similar expectations and view on the child-rearing process (Stewart et al., 1999; Yeh and Bedford, 2004; Ali and Frederickson, 2011; Tuli, 2012). As one of the fastest developing countries in Asia, Malaysia is currently at a crossroad whereby globalization led to the westernization and individualization of values which also intersect with strong traditional values and cultural expectations among each ethnic group. Thus, it can be predicted that parenting styles from the western societies may not be appropriate for the Malaysian family context as Malaysian parents may adopt a more localized and culturally specific parenting style. As reviewed, the impact of parenting style can vary across countries, societies, and cultures (Sangawi et al., 2015; Martinez et al., 2020). It is thus widely viewed that guan as an Asian parenting construct may be a unique parenting behavior to contribute to the development of filial piety belief among Malaysian adolescents.

It has commonly been assumed that the mother, as the main caregiver, may contribute more significantly to child upbringing than the father. However, Li and Lamb (2015) revealed the rise of paternal involvement in the child-rearing process. For many years, the finding on the simultaneous effect of paternal and maternal roles in child development is inconsistent and yet to be fully discovered (Putnick et al., 2012; Chen et al., 2016; Nijssens et al., 2020). A past study found that perceived maternal guan is higher than paternal guan among early adolescents; while only a high level of maternal guan significantly contributes to adolescent development (Lan et al., 2019). There is an apparent lack of study to identify the different role of paternal and maternal guan in adolescent development (Fok and Shek, 2011; Lan et al., 2019). As each parent might mediate adolescent development in a different way (Wang and Supple, 2010), this study aimed to investigate the respective relations of paternal and maternal guan with the developmental outcomes of adolescents.

Guan parenting and filial piety derived from Confucianism are the core values of Asian families. Guan that involves training may promote the development of filial piety belief among adolescent children. Chao (2000), who first introduced the concept of guan parenting, also further discussed the parents who endorse a high level of guan that emphasizes filial piety as a cultural notion in the family. It is believed that parents who perform guan tend to promote expected social behavior and moral values (i.e., loyalty to family and respect to elderly) and encourage conformity of children that could in turn instill the filial piety belief of children.

### Filial Piety

Filial piety or “xiao,” a customarily practiced family virtue that originated from Confucian teachings, has been a guiding principle for the younger generations on how to treat their parents and elderlies in the families (Wong et al., 2010; Woo, 2020). In the current society, the amalgamation of modern lifestyle and traditional value systems demonstrated that filial piety is no longer a pure Chinese notion but persists as a psychological concept that focuses on intergenerational relations. Filial piety has evolved as a part of Asian culture in maintaining family harmony (Carol, 2017).

Yeh and Bedford (2003) integrated the classic filial piety principle from Confucian teaching with current needs and constructed a dual-factor model of filial piety with two attributes namely reciprocal filial piety (RFP) and authoritarian filial piety (AFP). RFP focuses on the children’s readiness to build emotionally close relationships with and care for their elders as a way of paying gratitude for raising them. Children portray respect and obedience to their parents using AFP to conform to the social norm. FP as a strong factor in maintaining good relationships and harmony within family members (Woo, 2020) may impact the personal and social development of individuals including their life satisfaction. Existing literature indicated that RFP strongly brings a positive impact on one’s contentment which in turn makes them satisfied with their life and living. However, the underlying mechanism of the influence of FP on life satisfaction is still disputed (Sun et al., 2019). Hence, the interplay between filial piety and life satisfaction shall not be disregarded.

### Guan and Dual Filial Piety Model

It is a widely held view that adolescents who perceived a higher level of guan parenting tend to perform a higher level of RFP to repay care, concern, and love from parents. Past research into filial piety found that young adults who internalized guan parenting have a higher tendency to obey their parents and repay them as they received care and support from parents (Wang and Supple, 2010; Chen et al., 2016). Choi et al. (2013) have described guan as the manifestation of affection and monitoring from parents. A qualitative study in fact revealed that a higher level of covert parental control in the form of parental guidance, coaching, and monitoring motivated a sense of family loyalty among Asian Chinese adolescents (Lam, 2003). Thus, guan parenting which emphasized guidance and control can promote reciprocal support from children to voluntarily repay the benevolent upbringing effort from parents. A longitudinal study by Chen et al. (2016) revealed that young adults who perceived their mothers and fathers as supportive and highly involved parents reported higher levels of RFP. Leung et al. (2017) also reported that a high level of maternal control increases the level of filial piety of adolescents.

Besides, guan parents may also cultivate children’s filial piety belief and absolute obedience through instilling the importance of adhering to family rules and parental authority (Kim and Wong, 2002). Past studies found that authoritarian parenting that provides strict guidance contributed to a high level of AFP of adolescent children (Chen, 2014); in addition, parents with high demands on child behavior promote children’s conformity and unconditional compliance. Adolescent children who reported parental devotion and investment are more likely to repay *via* respecting and supporting parents as well as striving their best to honor the family. For example, Leung and Shek (2018) showed that a high level of paternal and maternal investment and selfless support positively predicted a higher level of AFP and RFP.

### Reciprocal Filial Piety and Life Satisfaction

Past studies among adolescents from Taiwan (Chen et al., 2016), Hong Kong (Leung et al., 2010; Chen, 2014), and China (Yeh et al., 2013; Sun et al., 2016) suggested that RFP positively contribute to their life satisfaction. This is further supported by recent research conducted among 716 school-going teenagers in Hong Kong that showed high RFP results in higher life satisfaction. The results were obtained upon controlling the sample’s age and perceived parental warmth (Leung and Shek, 2018). The significant positive associations between RFP and life satisfaction among adolescents were rationalized by the pleasure one experiences after fulfilling some payback to a person whom they owe. Consequently, the pleasure they experience results in higher satisfaction in their life. Healthy intergenerational communication not only improves RFP but also enhances interpersonal skills, for instance, empathy and self-disclosure. Subsequently, it provides a form of happiness to the individuals which includes an increase in their life satisfaction (Chen et al., 2018).

### Authoritarian Filial Piety and Life Satisfaction

Unlike RFP, AFP demands the suppression of a child’s desire to obey parents, the seniors, and family customs. AFP explains the need to fulfill parents’ wishes to maintain the family’s reputation. As the family is highly valued in Asian culture, many conform to the social norm to bring glory to their parents as it is associated with family status.

Results from previous studies on the effect of AFP on life satisfaction are inconclusive. For instance, a multinational study conducted in Hong Kong, Macau, and Taiwan reported that the samples which include teenagers and young adults practiced both RFP and AFP; however, their impacts on life satisfaction differ. AFP has a significant positive correlation with students’ life satisfaction in Taiwan and Hong Kong but not in Macau. Only RFP contributed to the life satisfaction of samples in Macau (Chen et al., 2019). On the other hand, a recent study conducted among 583 multi-ethnic adolescents in Malaysia showed a moderate positive association between both RFP and AFP with adolescents’ life satisfaction (Tan et al., 2018;Yan and Chen, 2018; Chen et al., 2019).

Some research with child samples reported a significant negative association between AFP and life satisfaction (Leung et al., 2010; Chen et al., 2019). Respondents who reported high AFP may need to compromise in many aspects for the sake of family harmony which in turn impacts their satisfaction toward life (Chen et al., 2019). The suppression of an individual’s desire to fulfill their obligation toward parents may result in lower self-worth (Leung et al., 2010), feelings of helplessness and frustration which subsequently reduces their satisfaction toward life (Yan and Chen, 2018; Chen et al., 2019). A study testing the association of AFP and life satisfaction was conducted among various age groups (children, adolescence, young adults), but the inconsistency of the findings further intrigues us to study the impact of AFP on one’s life satisfaction.

### Mediating Role of Filial Piety

Although guan parenting styles have been a widely studied topic in the field of parenting (Wu, 2012; Choi et al., 2013; Ang and Sin, 2021), the potential mechanism to explain the link between guan parenting and life satisfaction is still uncertain. Guan parenting and FP are closely affiliated as both originated from the traditional Chinese family socialization system. Guan parenting as the essential parental socialization element in Asian families may internalize filial piety values and promote better developmental outcomes for adolescent children.

Meanwhile, the association of guan parenting, filial piety, and also life satisfaction has been understudied in previous research. Filial piety might be one of the value aspects that linked guan parenting and life satisfaction (Yeh et al., 2013; Hsu, 2019). In a study by Wang and Supple (2010), a high level of guan parenting may mitigate adolescent depression *via* the internalization of filial piety belief as a feeling of filial devotion. However, this study also reported guan parenting in that high control can contribute to better academic achievement but not significantly reduce adolescent depression *via* filial piety. As previous studies found inconsistent findings to explain the relations between parenting behavior, filial piety, and adolescent well-being, this study aimed to investigate the mediating role from two aspects of filial piety (i.e., authoritarian and reciprocal filial piety) in the relation between parenting behavior and life satisfaction. This proposed idea is supported by the transactional model of development (Sanson et al., 2018) and the dual filial piety model (DFPM; Yeh and Bedford, 2003). The transactional model of development highlights the influential role of parents’ child-rearing belief on child positive development outcomes. It may explain the adolescents’ perceived life satisfaction that could be affected by their perception of parent–child interaction. On the other hand, DFPM posits that the two core filial piety values consist of potential benefits and risks to children’s psychological development (Leung et al., 2010). By integrating the propositions of these two models, a high level of guan parenting is implied to promote adolescents’ life satisfaction and the path may be varied by the mediating roles of AFP and RFP. Therefore, our study hypothesized that filial piety (AFP and RFP) acts as a mediator in the relationship between maternal and paternal guan parenting with life satisfaction among adolescents in Malaysia.

## Materials and Methods

### Participants

The respondents of this study consisted of 606 school-going adolescents selected from three states (Penang, Selangor, and Negeri Sembilan) in Peninsular Malaysia. A total of 12 secondary schools participated and were selected *via* the multistage cluster sampling method. The mean age of the respondents was 15.07years old with a standard deviation of 1.03. More than half of the respondents are females (52.1%). The respondents consist of 47.0% Malays, 27.6% Chinese, 23.6% Indians, and 1.8% Others (i.e., Dusun, Iban, Serani, and Thai).

### Procedure

Data collection approvals were obtained from the ministry of education, malaysia (MOE), the department of education (JPN) from each selected state, and school principals. Respondents were provided with information about the study, and parental consent was obtained before data collection. During the data collection day, the research team briefed all the respondents about the study’s background and objectives, reassured the privacy and confidentiality of their responses, their rights, and benefits of participation as well as potential risks of participation. The questionnaire and procedure of data collection were reviewed and approved by the Institutional Scientific and Ethical Review Committee.

### Measures

Paper-and-pencil questionnaires were prepared in the Malay language as the Malay language is the national language and medium of instruction in all the national schools in Malaysia. A backtranslation procedure was applied to translate the original English language questionnaire to the Malay language.

Filial piety scale (FPS; Yeh and Bedford, 2003) was used to measure adolescents’ filial piety. This scale consists of 16 items with eight items for each subscale on reciprocal filial piety and authoritarian filial piety. Respondents were required to respond on a 6-point Likert scale ranging from 1 (strongly disagree) to 6 (strongly agree). A mean score was computed where a higher score corresponds to higher filial piety. Sample item for reciprocal filial piety was “Be grateful to my parents for raising me,” and sample item for authoritarian filial piety was “Take my parents’ suggestions even when I do not agree with them.” The Cronbach alpha for the scales was 0.79 for reciprocal filial piety and 0.70 for authoritarian filial piety, respectively.

Satisfaction with life scale (SWLS; Diener et al., 1985) was used to measure global cognitive judgment of satisfaction with one’s life. SWLS consists of five items on a 7-point scale ranging from 1 (strongly disagree) to 7 (strongly agree). A mean score computed with a higher score indicates higher life satisfaction. The Cronbach alpha of 0.83 was reported in this study.

Guan parenting scale (Chao, 1994) was used to assess adolescents’ perceived parenting behavior. This scale focuses on the aspects of Chinese parenting which reflect “training” literally referring to parental control in cultivating expected social values and guiding appropriate behavior of children (Chao, 1994). This scale consists of eight items for the respective paternal and maternal guan perspectives. Respondents were requested to respond on a scale ranging from 1 (strongly disagree) to 5 (strongly agree). A mean score computed with a higher score indicates a higher guan parenting. A sample item was “Mother/father presents his/her expectations to me.” The Cronbach alpha for the scales was 0.85 for Father guan and 0.80 for Mother guan in this study.

### Data Analysis Plan

The results of this study were analyzed using IBM SPSS version 22. The data were first computed to examine the association between guan parenting, filial piety, and life satisfaction using Pearson’s product–moment correlation. The hypothetical mediating model was tested using the SPSS macro-PROCESS (Hayes, 2013) with bias-corrected bootstrap confidence interval (CI) based on 5,000 bootstrapped samples. All the mediation analyses treated adolescent sex and ethnicities as covariates. The indirect effect was considered statistically significant if the CI does not contain zero. There were no missing data detected in this dataset.

## Results

Table 1 presents the correlation results between variables of this study. Results found guan parenting (i.e., paternal and maternal) positively associated with filial piety (i.e., reciprocal and authoritarian). Results also revealed that adolescents’ life satisfaction positively linked with guan parenting and filial piety sub-scales. Thus, the results offered support to our hypothesis.

**Table 1:** Correlation analysis between variables.

	2	3	4	5
1. Maternal parenting	0.76^***^	0.23^***^	0.32^***^	0.09^*^
2. Paternal parenting	–	0.23^***^	0.34^***^	0.13^**^
3. Reciprocal FP		–	0.55^***^	0.38^***^
4. Authoritarian FP			–	0.35^***^
5. Life satisfaction				–

*** *p<0.001,*

** *p<0.01,*

* *p<0.05.*

### The Mediating Role of Reciprocal and Authoritarian Filial Piety

**Figure 1:**
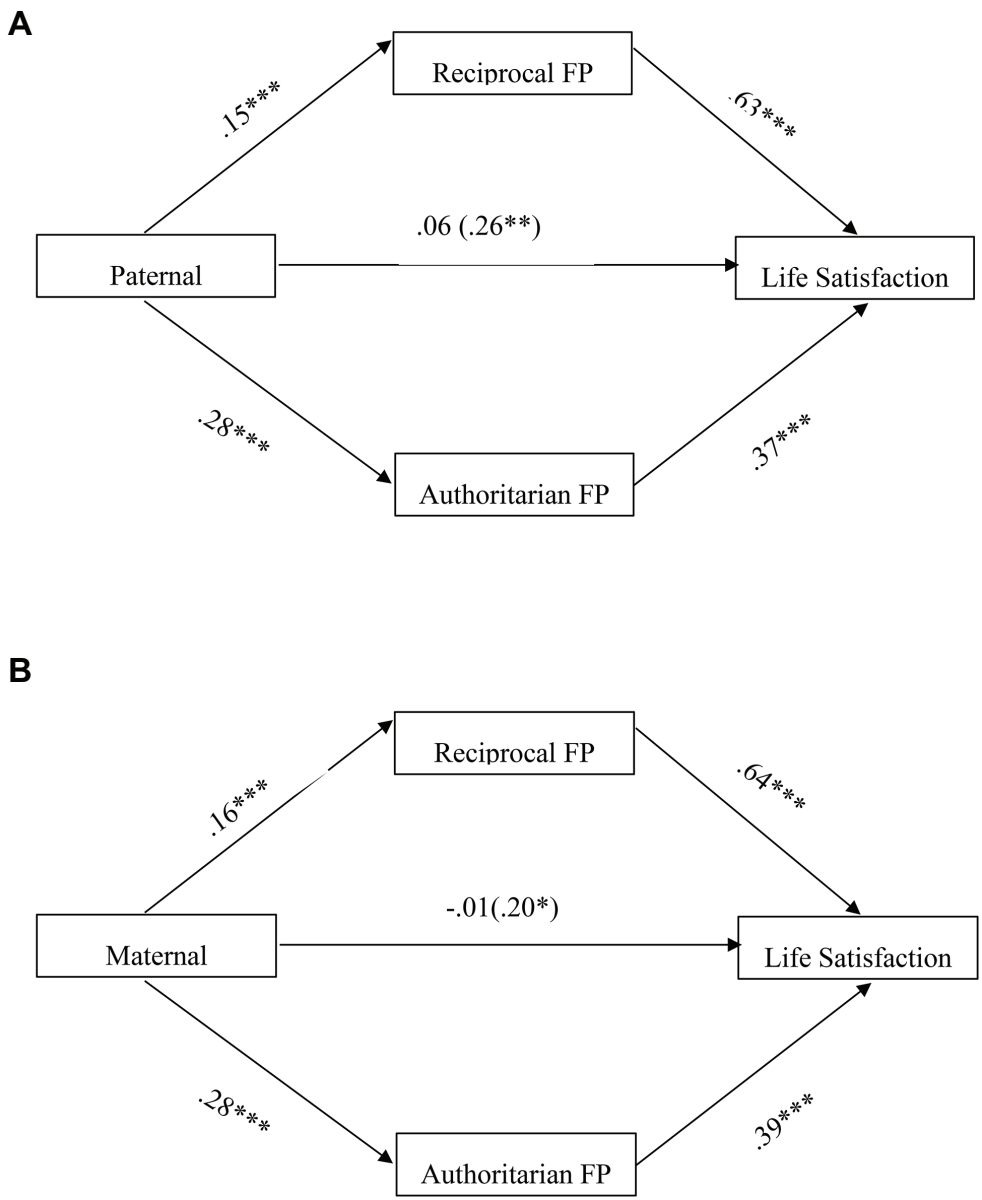
**(A)** Mediation model showing the effect of paternal parenting and filial piety on life satisfaction. The values shown are unstandardized coefficients. The total effect of paternal parenting was shown in parenthesis. ^***^p<0.001; ^**^p<0.01. **(B)** Mediation model showing the effect of maternal parenting and filial piety on life satisfaction. The values shown are unstandardized coefficients. The total effect of maternal parenting was shown in parenthesis. ^***^p<0.001.

Figure 1A, Table 2 present the findings for the hypothesized indirect effect of paternal guan parenting on the adolescents’ life satisfaction *via* reciprocal and authoritarian FP. The findings indicated that paternal guan parenting was positively associated with adolescents’ life satisfaction (*B*=0.26, *t*=3.93, *p*=0.001), reciprocal FP (*B*=0.15, *t*=5.09, *p*<0.001), and authoritarian FP (*B*=0.28, *t*=7.64, *p*<0.001) after controlling the effects of sex and ethnicities. Positive relationships were also found between life satisfaction with reciprocal FP (*B*=0.63, *t*=6.43, *p*<0.001) and authoritarian FP (*B*=0.37, *t*=4.83, *p*<0.001). The association between paternal guan parenting and life satisfaction became not significant (*B*=0.06, *t*=1.00, *p*=0.317) after controlling the effects of reciprocal and authoritarian filial piety as well as the covariates (i.e., Chinese, *B*=0.52, *t*=5.02, *p*<0.001). As expected, the mediating effect of reciprocal filial piety, *B*=0.09, SE=0.02, 95% CI [0.05, 0.14] and authoritarian filial piety, *B*=0.10, SE=0.03, 95% CI [0.06, 0.16] were both found to be significant for the association between paternal guan parenting and life satisfaction.

**Table 2:** Summary of the mediation model results.

	*B*	SE	*t*	95% CI
Paternal				
Pat → LS (total)	0.26^**^	0.07	3.93	[0.13, 0.23]
Pat → LS (direct)	0.06	0.06	1.00	[−0.06, 0.19]
Pat → RFP	0.15^***^	0.03	5.09	[0.09, 0.20]
RFP→LS	0.63^***^	0.10	6.43	[0.09, 0.20]
Pat → AFP	0.28^***^	0.04	7.64	[0.21, 0.35]
AFP→LS	0.37^***^	0.08	4.83	[0.09, 0.20]
Indirect effect (Total)	0.20	0.04		[0.13, 0.27]
Indirect effect (RFP)	0.09	0.02		[0.04, 0.14]
Indirect effect (AFP)	0.10	0.03		[0.06, 0.16]
Indirect effect (RFP - AFP)	−0.01	0.04		[−0.09, 0.06]
Maternal				
Mat → LS (total)	0.20^**^	0.07	2.67	[0.05, 0.34]
Mat → LS (direct)	−0.01	0.07	−0.21	[0.15, 0.12]
Mat → RFP	0.16^***^	0.03	4.97	[0.10, 0.22]
RFP→LS	0.64^***^	0.10	6.49	[0.45, 0.83]
Mat → AFP	0.28^***^	0.04	6.83	[0.20, 0.36]
AFP→LS	0.39^***^	0.08	5.13	[0.24, 0.54]
Indirect effect (Total)	0.21	0.05		[0.13, 0.30]
Indirect effect (RFP)	0.10	0.03		[0.05, 0.17]
Indirect effect (AFP)	0.11	0.03		[0.06, 0.17]
Indirect effect (RFP - AFP)	−0.01	0.04		[−0.09, 0.07]

*Pat*, *Paternal parenting, Mat, Maternal parenting, LS, Life satisfaction, RFP, Reciprocal filial piety, AFP, Authoritarian filial piety, total, total effect, direct*, *direct effect, CI, 95% bias-corrected confidence interval.*

*** *p<0.001,*

** *p<0.01.*

Besides, almost similar results were obtained for the association among maternal guan parenting, reciprocal and authoritarian FP, and life satisfaction (see Figure 1B). There was a positive linkage between maternal guan parenting with life satisfaction (*B*=0.20, *t*=2.67, *p*=0.008), reciprocal filial piety (*B*=0.16, *t*=4.97, *p*<0.001) and authoritarian filial piety (*B*=0.28, *t*=6.83, *p*<0.001). Meanwhile, life satisfaction was associated positively with reciprocal filial piety (*B*=0.64, *t*=6.49, *p*<0.001) and authoritarian filial piety (*B*=0.39, *t*=5.13, *p*<0.001). The association between maternal guan parenting and life satisfaction became not significant, *B*=−0.01, *t*=−0.21, *p*=0.835 after controlling the effects of both reciprocal and authoritarian filial piety and the covariates (i.e., sex, *B*=0.17, *t*=1.99, *p*=0.047; Chinese, *B*=0.49, *t*=4.68, *p*<0.001). The indirect effect of maternal guan parenting on life satisfaction *via* reciprocal filial piety was found to be significant, *B*=0.10, SE=0.03, 95% CI [0.05, 0.17]. The mediating effect of authoritarian filial piety, *B*=0.11, SE=0.03, 95% CI [0.06, 0.17], was significant in the association between maternal parenting and life satisfaction (refer to Table 2).

## Discussion

The present study proposed and tested two parallel mediation models to clarify the relationships among (paternal and maternal) Guan parenting, reciprocal filial piety (RFP), authoritarian filial piety (AFP), and life satisfaction among adolescents in Malaysia. The results support that both paternal and maternal Guan parenting have an indirect relationship with life satisfaction through RFP and AFP, respectively.

Paternal and maternal Guan parenting were examined in the present study to address the necessity of understanding the unique role of father and mother in adolescent development (Fok and Shek, 2011; Lan et al., 2019). Both types of Guan parenting were found to have a positive relationship with life satisfaction. The result is consistent with past findings in that adolescents who received Guan parenting tend to report a higher level of well-being (e.g., Stewart et al., 2002). Moreover, in line with past studies (e.g., Leung et al., 2010; Chen, 2014), both paternal and maternal Guan parenting are positively associated with RFP and AFP, respectively. In other words, for parents who employ Guan parenting, their children tend to perceive the obligation of paying back to and obeying them. Taken together, the results imply that Guan parenting practiced by a father or mother is beneficial to adolescents’ filial piety and life satisfaction. Note that, although traditionally the mother is the primary caregiver who plays a dominant role in children’s development, our findings are not uncommon. Indeed, some studies have shown that fathers also play an important role in children’s socioemotional and behavioral development (e.g., Cabrera et al., 2014; Parke and Cookston, 2019; Sifaki et al., 2020). For instance, Gao et al. (2021) collected data from 466 Chinese middle and high school students using a longitudinal design with an 8-month interval. Consistent with the findings from maternal parenting styles, Gao and colleagues found that paternal emotional warmth reported at Time 1 had a positive relationship, while paternal harsh discipline measured at Time 1 had a negative relationship, with life satisfaction reported at Time 2.

Consistent with the findings derived from college students in Taiwan (Yan and Chen, 2018; Chen et al., 2019), both RFP and AFP were found to have a positive relationship with life satisfaction in the present study. While the results indicate that both types of filial piety matter to our participants, it is noteworthy that the RFP showed a stronger relationship with life satisfaction than AFP. The result could be due to children having to compromise their desires or plans to meet the needs of the family when practicing AFP. Such suppression may reduce self-worth (Leung et al., 2010) and satisfaction toward life (Chen et al., 2019).

Overall, the present study expands the literature by offering empirical support for indirect relationship between paternal and maternal Guan parenting with life satisfaction through both reciprocal and authoritarian filial piety, respectively. The mediation model not only provides insights into the underlying mechanism of the beneficial relationship between Guan parenting and adolescents’ life satisfaction but also serves as a baseline for future researchers to develop a theoretical framework of Guan parenting and adolescents’ well-being. For instance, future researchers may collect qualitative data and then use the grounded theory approach to develop a theory to explain the role of Guan parenting in adolescents’ well-being.

Although the results are promising, we acknowledge that the mediation model is not saturated and can be further expanded. For example, it is important to know why Guan parenting is beneficial to filial piety and whether the positive relationship is conditional on adolescents’ attitudes toward Guan parenting. In the same vein, future researchers are suggested to collect data on parenting from fathers and mothers, instead of measuring adolescents’ perceived parenting, to avoid the high correlation between paternal and maternal parenting scores. Moreover, the present study is unable to demonstrate the causal relationship among the variables with a cross-sectional design. However, it does not seem reasonable to assume that life satisfaction precedes Guan parenting and filial piety. Similarly, filial piety perceived by adolescents is less plausible to be an antecedent factor of parents’ child-rearing techniques. This is because parenting styles have been found to shape filial piety (e.g., Chen, 2014; Chen et al., 2016) but not vice versa. Finally, the data of the present study were collected from three states (out of the 13 states and 3 federal territories) in Malaysia using the multistage cluster sampling method. The generalizability of the findings to other states and cultural contexts remains open. Future researchers are thus recommended to replicate the present study in other states of Malaysia or even other countries.

## Conclusion

Parental and maternal Guan parenting are indirectly and positively associated with adolescents’ life satisfaction through reciprocal and authoritarian filial piety, respectively. The findings highlight that this unique Asian parenting style is beneficial. Researchers in future studies are warranted to further explore the positive role of this Asian parental control.

## Data Availability Statement

The raw data supporting the conclusions of this article will be made available by the authors, without undue reservation.

## Ethics Statement

The studies involving human participants were reviewed and approved by Scientific and Ethical Review Committee of Universiti Tunku Abdul Rahman, Malaysia. Written informed consent to participate in this study was provided by the participants’ legal guardian/next of kin.

## Author Contributions

SN and S-WG have made a considerable contribution to the concept or design of the article. All authors were largely involved in the acquisition of the data. S-AT and C-ST were involved in the analysis and interpretation of data for the article. All authors drafted the article, and J-PT revised it critically for important intellectual content. Upon several revision, all authors approved the version to be submitted and agreed to be accountable for all aspects of the work related to the accuracy or integrity of any part of the work.

## Funding

This study was funded by Universiti Tunku Abdul Rahman Research Fund [IPSR/RMC/UTARRF/2014-C2/T04].

## Conflict of Interest

The authors declare that the research was conducted in the absence of any commercial or financial relationships that could be construed as a potential conflict of interest.

## Publisher’s Note

All claims expressed in this article are solely those of the authors and do not necessarily represent those of their affiliated organizations, or those of the publisher, the editors and the reviewers. Any product that may be evaluated in this article, or claim that may be made by its manufacturer, is not guaranteed or endorsed by the publisher.
